# Seasonal distribution and environmental parameters associated with *Brugia pahangi* and *Dirofilaria immitis* in naturally infected dogs in Bangkok and vicinity, Thailand

**DOI:** 10.1038/s41598-021-84215-8

**Published:** 2021-02-25

**Authors:** Wanarit Jitsamai, Pimsiri Piromkij, Patchana Kamkong, Sudchit Chungpivat, Piyanan Taweethavonsawat

**Affiliations:** 1 Parasitology Unit, Department of Pathology, Faculty of Veterinary Science, Chulalongkorn University, Bangkok, 10330 Thailand; 2 Hato Pet Wellness Centre, Trail and Tail, Bangkok, 10110 Thailand; 3 Vet Central Lab, Limited Partnership, Nonthaburi, 11000 Thailand

**Keywords:** Parasite biology, Parasite development, Infectious diseases

## Abstract

*Dirofilaria immitis* and *Brugia pahangi* are vector-borne parasites found in dogs and cats, including Thailand. In order to evaluate the effects of season and environmental parameters on the prevalence of these parasites, this retrospective study was conducted in 2019. A total of 79,506 canine blood samples were examined. *B. pahangi* was found in 0.55% of samples (438/79,506; 95% CI 0.50–0.61) while *D. immitis* was detected in 0.43% (345/79,506; 95% CI 0.39–0.48). One-way ANOVA found no effect of seasonal conditions on prevalence. For *B. pahangi*, the parameters rainfall, relative humidity and sunshine hours showed associations with p ≤ 0.20 and were included in multiple logistic regressions resulting in adjusted odds ratios of 0.53, 1.31 and 0.55, respectively. For *D. immitis,* only average temperature showed p ≤ 0.20, resulting in an odds ratio of 0.42. In conclusion, Thailand has environmental parameters that do not change very much during the year, so they might not affect the prevalence of two filarial nematodes. However, the threat of *B. pahangi* and *D. immitis* should not be ignored, especially in subtropical regions where their vectors are abundant. Both owners and veterinarians should be concerned about filarial prevention and control of *D. immitis* and *B. pahangi*.

## Introduction

*Dirofilaria immitis* and *Brugia pahangi* are vector-borne parasites in dogs and cats that have zoonotic potential and are common in tropical, subtropical and some temperate regions of the world, including Thailand^1^. *D. immitis* is well known as a causative agent of heartworm disease in dogs and cats^2,3^. Microfilariae of the dog heartworm *D. immitis* present a subperiodicity without clear nocturnal or diurnal peaks, where a wave pattern is apparent but microfilaria do not completely disappear from the peripheral blood. However, the physiological periodicity is unknown^4^. It is also known as an occasional cause of pulmonary dirofilariasis in humans^5^. Lymphatic filariasis, a neglected tropical disease that is caused by filarial nematodes in the genus *Brugia*, affects approximately 80 countries around the world, particularly in dogs, cats and humans^6^.

Mosquitoes in the genera *Mansonia*, *Armigeres* and *Aedes* are potential vectors of dirofilariasis and lymphatic filariasis^7^. The tropical atmosphere is the most suitable for these mosquito vectors to survive. Increasing global temperatures and humidity are advantageous to the spread of mosquitoes and are also enhancing the effectiveness of pathogen transmission through mosquito-borne diseases such as dengue, malaria and lymphatic filariasis^8^. For instance, there is some evidence indicating that the prevalence of mosquito-borne disease in South America and South-East Asia relates to the El-Niño phenomenon. The relationship between El-Niño and the increasing risk of these diseases can be attributed to the rise in global temperature^9–11^.

Adults of *D. immitis* reside in the pulmonary artery and can induce endothelial damage. Some cases may develop into canine eosinophilic pulmonary granulomatosis caused by the infiltration of eosinophils^12^. The severity of heartworm disease depends on the number of adult worms, the duration of infection and the host immune response. Adult heartworms can release vasoactive substances that result in vasoconstriction and hypoxia, which lead to pulmonary hypertension, and antigens may pass through to the lung causing eosinophilic pneumonitis^13^. Chronic infection leads to retrograde migration of adults to the right atrium and vena cava causing deflection of the tricuspid valve, resulting in clinical signs of right-sided heart failure^14^. *B. pahangi* infection can manifest in four ways: (1) no clinical signs with no microfilaremia; (2) no clinical signs with microfilaremia; (3) acute short duration lymph node enlargement and/or limb oedema with microfilaremia; and (4) chronic limb oedema without microfilaremia^15^. Experimentally infected dogs showed abscesses in the adipose connective tissue around the popliteal node and nerve-cell tumours near the sciatic nerve. In one dog, lymphatic ducts were dilated distal to the popliteal node^16^. Dogs demonstrated a range of clinical signs, including episodic lymphadenopathy, lymphangitis, and limb oedema similar to the clinical signs reported in humans^17^.

Only a few studies performed in South-East Asian countries have reported the incidence and the population of animals affected by dirofilariasis and lymphatic filariasis^18–20^, and no studies have been designed to monitor the environmental factors affecting the distribution of these diseases. This study evaluated the association between seasonal and environmental factors related to the prevalence of *D. immitis* and *B. pahangi* infections in domestic dogs in Bangkok, Thailand and its vicinity.

## Results

*Brugia pahangi* was found in 0.55% (438/79,506; 95% CI 0.50–0.61) and *D. immitis* was detected in 0.43% (345/79,506; 95% CI 0.39–0.48) of 79,506 samples tested during January to December 2019. The monthly detection rate of *B. pahangi* and *D. immitis* is shown in Table 1. The prevalence of *B. pahangi* infection was higher than that of *D. immitis* in all months.

**Table 1:** Prevalence of *B. pahangi* and *D. immitis* positive rate during January–December 2019.

Months	*B. pahangi* (%)	95% CI	*D. immitis *(%)	95% CI	N
January	0.42	0.28–0.60	0.11	0.25–0.55	6925
February	0.64	0.46–0.86	0.16	0.33–0.68	6448
March	0.69	0.51–0.91	0.11	0.22–0.51	6960
April	0.52	0.35–0.74	0.10	0.22–0.54	5965
May	0.53	0.37–0.73	0.17	0.35–0.70	6777
June	0.50	0.35–0.69	0.08	0.22–0.51	7002
July	0.53	0.37–0.72	0.15	0.28–0.59	7219
August	0.48	0.33–0.67	0.14	0.32–0.66	7057
September	0.57	0.41–0.78	0.18	0.40–0.76	7000
October	0.39	0.26–0.57	0.16	0.36–0.71	6838
November	0.69	0.49–0.93	0.15	0.33–0.71	5826
December	0.71	0.51–0.97	0.12	0.27–0.54	5489

**Figure 1:**
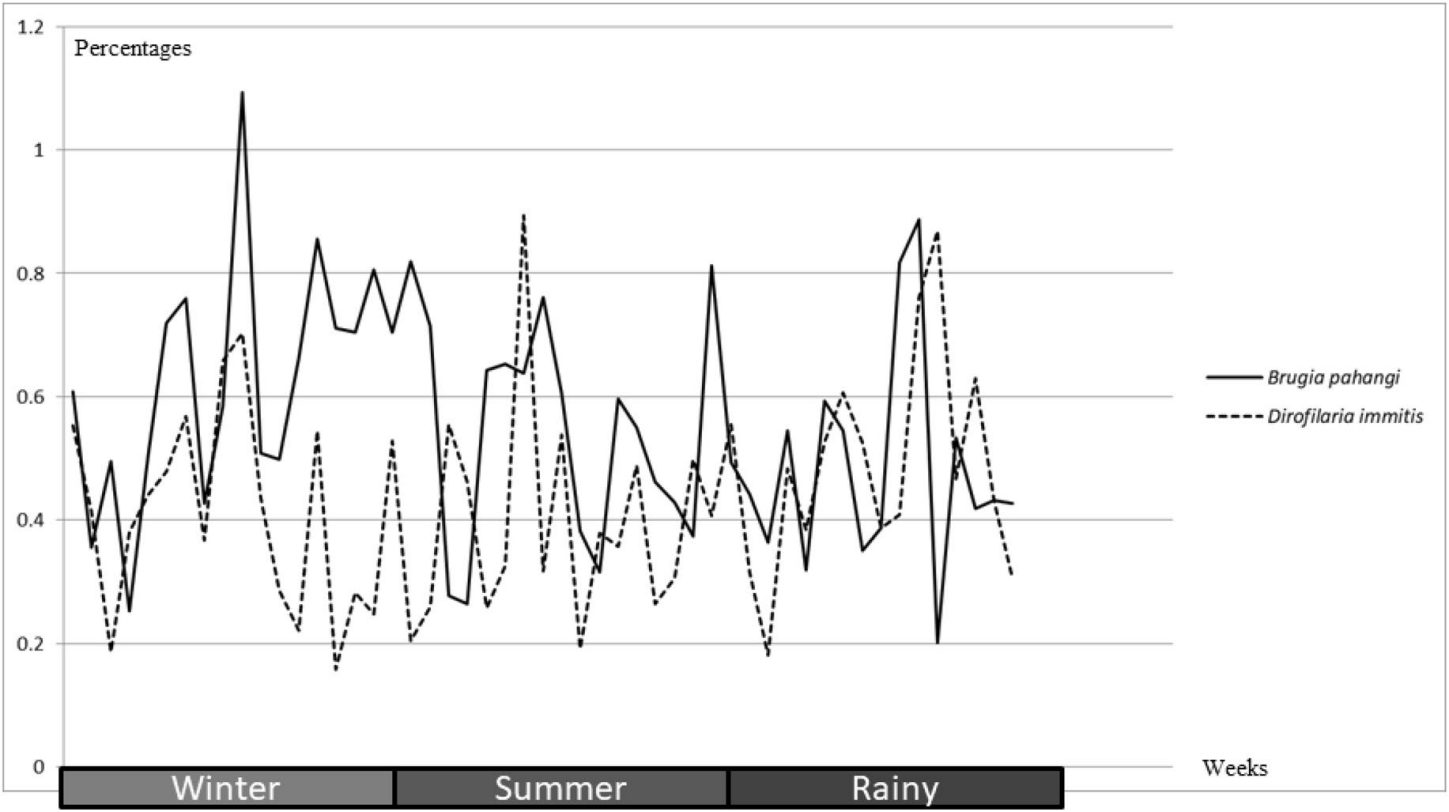
Line chart demonstrated positive rates of *B. pahangi* and *D. immitis* by seasons.

The prevalence by season is shown in Fig. 1. The year was divided into three seasons; winter (November, December, January and February), summer (March, April, May and June) and the rainy season (July, August, September and October). The association between season and prevalence was not significant for either *B. pahangi* (p = 0.23) or *D. immitis* (p = 0.09).

**Figure 2:**
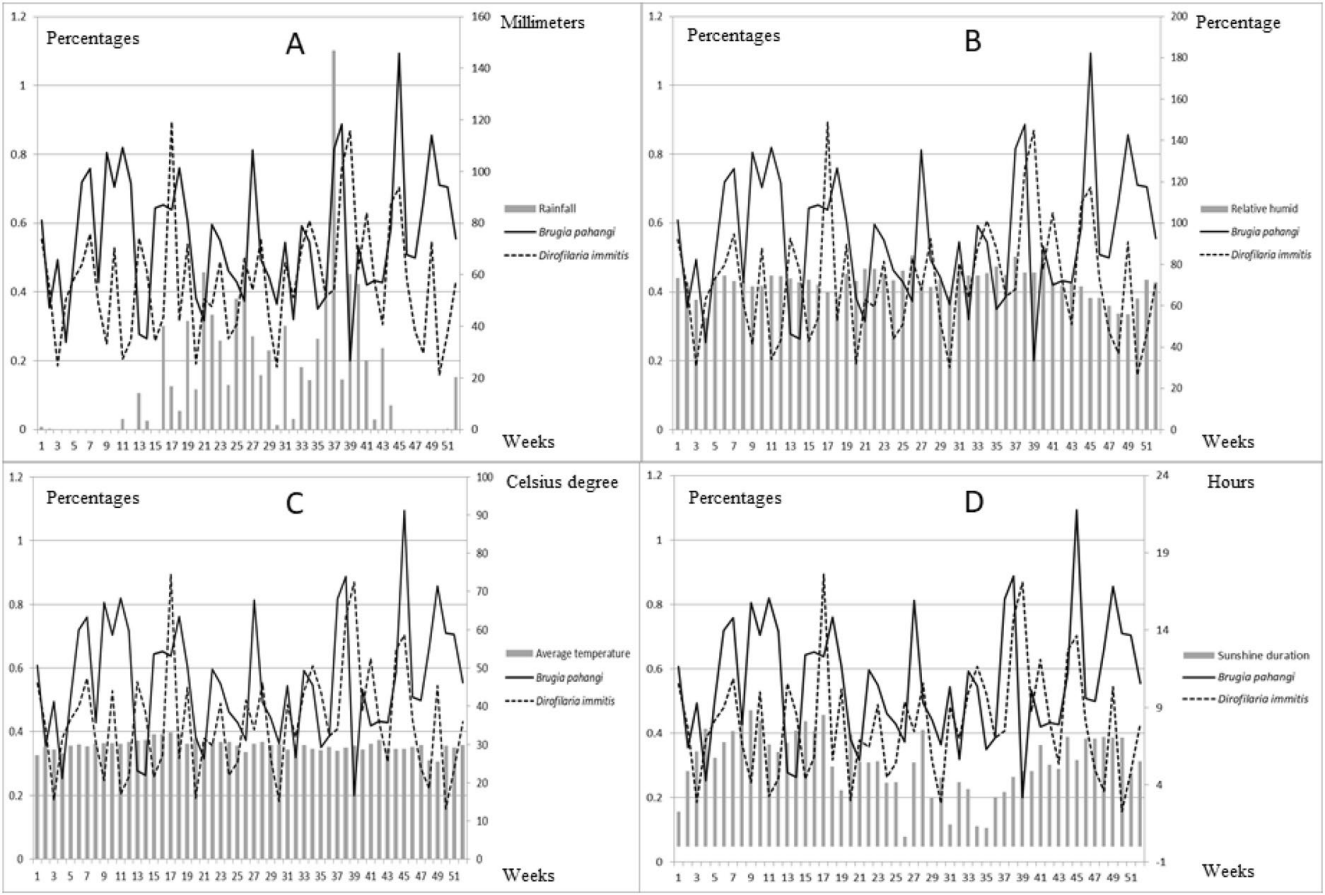
Line chart (*B. pahangi and D. immitis*) revealed positive of each filarial by week and bar chart demonstrate parameters including rainfall (**A**), relative humidity (**B**), average temperature (**C**) and sunshine duration (**D**).

The environmental parameters related to season, including rainfall, relative humidity, average temperature and sunshine duration, were selected based on vector and parasite biology. These parameters along with infection rates of *B. pahangi* and *D. immitis* are shown in Fig. 2.

Environmental factors, including rainfall, relative humidity, average temperature and sunshine duration, were analysed to reveal associations between infection rates and each factor using Pearson’s correlation. The results revealed no significant relation for any parameter, as shown in Table 2; hence, no parameter had a significant effect and linear regression was not performed.

**Table 2:** Showed the correlation of environmental parameters between *B. pahangi* and *D. immitis* using Pearson’s correlation.

Parameters	*B. pahangi*			*D. immitis*		
	r value	95% CI	p value	r value	95% CI	p value
Rainfall	− 0.11	− 0.37–0.17	0.43	0.15	− 0.13–0.41	0.27
Sunshine duration	0.19	− 0.09–0.44	0.19	− 0.11	− 0.38–0.17	0.43
Relative humidity	− 0.15	− 0.41–0.13	0.30	0.19	− 0.09–0.44	0.19
Average temperature	− 0.07	− 0.35–0.20	0.59	− 0.06	− 0.33–0.22	0.69

Since continuous data analysis using Pearson’s correlation could not describe the association, categorical data analysis was performed by transforming the data using the average of each parameter as the cutoff: 20.38 mm rainfall, 29.78 °C average temperature, 5.52 h sunshine duration and 71.35% relative humidity, and the prevalence of *B. pahangi* 0.55% and *D. immitis* 0.43%. Categorical analyses including crude odds ratio and 95% CI are shown in Tables 3 and 4. The parameters showing p ≤ 0.20 were included in multiple logistic regression, which was used to calculate the adjusted odds ratio.

**Table 3:** Univariates and multivariable logistic regression demonstrate association between *B. pahangi* infection and environment parameters.

Parameters	Crude odds ratio	95% CI	p value	Adjusted odd ratio
Rainfall	0.41	0.12–1.34	0.14*	0.53
Sunshine duration	2.07	0.67–6.38	0.20*	1.31
Relative humidity	0.44	0.14–1.39	0.16*	0.55
Average temperature	1.09	0.36–3.29	0.88	ND

*ND* not determined, *p > 0.20.

**Table 4:** Univariates and multivariable logistic regression demonstrate association between *D. immitis* infection and environment parameters.

Parameters	Crude odds ratio	95% CI	p value	Adjusted odd ratio
Rainfall	1.26	0.40–3.93	0.69	ND
Sunshine duration	1.48	0.49–4.48	0.48	ND
Relative humidity	1.56	0.58–1	0.45	ND
Average temperature	0.42	0.14–1.28	0.12	ND

*ND* not determined, *p > 0.20.

The results revealed that only average temperature showed an association with *D. immitis* infection (p ≤ 0.20). However, three parameters were included in the logistic regression in order to estimate associations between environmental parameters and *B. pahangi* infection. The multicollinearity between relative humidity and rainfall was checked using the Chi-square test and the results revealed no multicollinearity between these parameters. Unfortunately, no parameter was significant using logistic regression. The adjusted odds ratio (OR) for each parameter associated with *B. pahangi* infection was as follows: rainfall 0.53, sunshine duration 1.31 and relative humidity 0.55.

## Discussion

The current study presents the prevalence of *B. pahangi* and *D. immitis* circulating in owned dogs in Bangkok and its vicinity in 2019. A large number of the samples were collected from diseased dogs, which may have influenced the prevalence recorded herein. On the other hand, no antigen detection studies were carried out to detect amicrofilaemic animals, and only those that had detectable microfilariae in blood were counted. A limitation of this study was that samples were obtained from a clinical laboratory, and thus we do not have any details about the infected dogs. In this study, the monthly prevalence of the two filarial nematodes shown in Table 1 revealed that *B. pahangi* was more common than *D. immitis*. According to veterinarians, owners protect their pets from heartworm disease by using commercial products comprising extra-label ivermectin and its derivatives to control ticks, and these measures have been reducing the prevalence of *D. immitis*. In 2003, Nithiuthai reported that 10.2% of samples contained microfilaria of *D. immitis* (n = 83,476) in Bangkok during 1999–2001^21^. Unfortunately, given the lack of a prevention programme for *B. pahangi*, there is no medication to prevent *B. pahangi* infection; one of the important outcomes of this study was that the prevalence of canine lymphatic filariasis is higher than canine heartworm disease.

Our analysis showed that prevalence was not influenced by the season. Interestingly, *D. immitis* was more prevalent than *B. pahangi* in weeks 17, 34 and 41. Thailand is in South-East Asia, which has a tropical savanna climate under the influence of the South Asian monsoon system. Some environmental parameters vary little throughout the year, including average temperature and humidity, thus making it difficult to associate these parameters with filarial nematode infection. There were no significant correlations between infection rate and these environmental parameters. However, sunshine duration seemed to show some correlation with *B. pahangi* infection rates (p = 0.19), as did relative humidity with that of *D. immitis* (p = 0.19). It was assumed that the longer daylight during summer affects some endocrine mechanisms in the dog, which may stimulate the female filarial worms to produce greater numbers of microfilariae^4^. Univariate analysis of *B. pahangi* prevalence revealed that three parameters showed p-values of ≤ 0.20: rainfall, relative humidity and sunshine duration, and so these were included in the multivariable logistic regression. Unfortunately, no parameter was significant in this regression; however, sunshine duration showed a positive adjusted OR of 1.31, which related to correlation. Vectors of most importance for filarial nematodes include *Aedes* spp. *Culex* spp. and *Anopheles* spp. and these are considered as potential vectors for *D. immitis*^22,23^, whereas *Armigeres* spp. and *Manosonia* spp. are considered as vectors for *B. pahangi* ^24^. The OR-values of rainfall associated with *B. pahangi* and *D. immitis* prevalence were interesting: that for *B. pahangi* was 0.41, indicating that the rains are a protective factor, by washing floating water plants out of swamp ponds and leading to a lack of suitable places for the life cycle of *Mansonia* spp.^25^, one of its potential vectors. On the other hand, the OR-value for *D. immitis* was 1.26, indicating that rain is a risk factor, due to clean water being suitable for *Aedes* spp.^23^, one of its potential vectors.

*Brugia pahangi* and *D. immitis* are not the only two filarial nematodes reported in Thailand; other filaria include *Brugia malayi*^26^ and *Dirofilaria repens*^27^. In this study, *D. repens* was found in one sample in December. Microfilaria of *D. repens* can be differentiated morphologically as they have two nuclei in the cephalic space. Molecular diagnosis is an alternative method for identification of *D. immitis* and *D. repens*.

## Conclusions

In conclusion, environmental parameters in Thailand do not change much during the year, so they might not affect the prevalence of *B. pahangi* and *D. immitis*. The prevalence of these two filarial nematodes should not be ignored, and owners and veterinarians should be educated in the prevention and control of filarial nematodes in order to decrease the prevalence of these neglected canine vector-borne diseases.

## Methods

### Study design and sample collection

This retrospective study was conducted between January and December 2019. The results of blood examination were provided by the Vet Central Lab, which collected samples from private veterinary clinics and animal hospitals around Bangkok and its vicinity. All environmental parameters, including rainfall, relative humidity, average temperature and sunshine duration were obtained from the information service of the Thai Meteorological Department, Ministry of Digital Economy and Society. The data were the average from four stations in Bangkok: the Queen Sirikit National Convention Center, Bangkok Port, Thai Meteorological Department Bang Na and Don Mueang International Airport.

### Blood examination and parasite identification

A total of 79,506 EDTA-anticoagulated blood samples were collected from owned dogs and submitted to the Vet Central Lab. Buffy-coat thin blood smears were performed and stained with Wright-Giemsa stain. The positive microfilariae were examined by light microscopy. Unsheathed and sheathed microfilaria were tested for acid phosphatase activity to identify species as *D. immitis*^28^ and *B. pahangi*^28^*,* respectively.

### Statistical analysis

The prevalence of filarial worms was demonstrated using descriptive statistics with a 95% confidence interval (95% CI). The association between filarial worm infection and environmental parameters was analysed as continuous data using Pearson’s correlation. Since one week was considered as a replicate, each parameter was represented as the average per week, except rainfall, which combined all data to represent one week. If these results were not acceptable, the infection rate and other continuous data were transformed using the average of each parameter or infection rate as the cutoff and analysed using the crude odds ratio at 95% CI. The parameters showing p ≤ 0.20 were checked for multicollinearity and included in multiple logistic regression. Statistical analysis used R software version 3.5.3.
